# Simple and Efficient Route toward Improved Energetics within the Framework of Density-Corrected Density Functional Theory

**DOI:** 10.1021/acs.jctc.3c00441

**Published:** 2023-08-01

**Authors:** Daniel Graf, Alex J. W. Thom

**Affiliations:** Yusuf Hamied Department of Chemistry, University of Cambridge, Cambridge CB2 1EW, United Kingdom

## Abstract

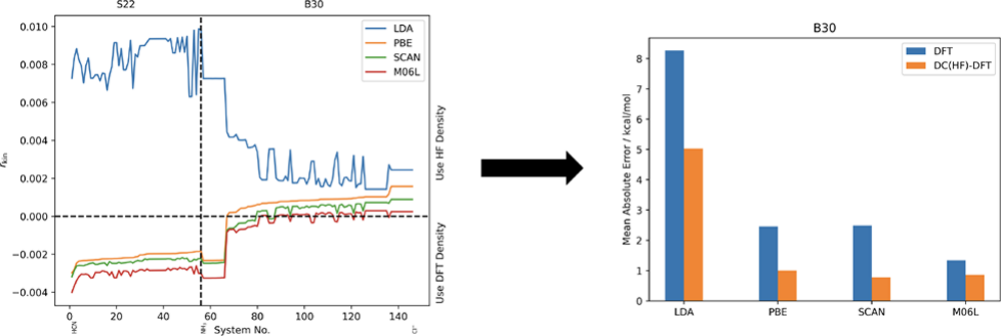

The crucial step in density-corrected Hartree–Fock density functional theory (DC(HF)-DFT) is to decide whether the density produced by the density functional for a specific calculation is erroneous and, hence, should be replaced by, in this case, the HF density. We introduce an indicator, based on the difference in noninteracting kinetic energies between DFT and HF calculations, to determine when the HF density is the better option. Our kinetic energy indicator directly compares the self-consistent density of the analyzed functional with the HF density, is size-intensive, reliable, and most importantly highly efficient. Moreover, we present a procedure that makes best use of the computed quantities necessary for DC(HF)-DFT by additionally evaluating a related hybrid functional and, in that way, not only “corrects” the density but also the functional itself; we call that procedure corrected Hartree–Fock density functional theory (C(HF)-DFT).

## Introduction

Density functional theory (DFT) is a widely used approach in computational physics and chemistry owing to the fact that it allows for the relatively simple approximation of many-body effects, providing useful accuracy at low computational cost. Despite the existence of hundreds of density functionals, most DFT calculations use only a few standard functionals, often in the form of (meta) generalized gradient approximations ((m)GGAs).^1^ While (m)GGAs are true Kohn–Sham^2^ (KS) density functionals, consisting of a local multiplicative KS potential, local and semilocal density functionals tend to overdelocalize charge. This overdelocalization is associated with several well-known problems in density functional theory, including delocalization error,^3−10^ one-electron self-interaction error,^11^ many-electron self-interaction error,^12−14^ missing derivative discontinuities in the energy as particle numbers pass through integer values—density functionals are too smooth—^15−17^ and fractional charge and spin errors,^18−20^ and is the reason for, e.g., unbound anions, incorrect molecular dissociation curves, and underestimated reaction barriers.^5,21^

To address the problem of overdelocalization, various approaches have been developed, such as self-interaction corrections,^11^ the admixture of exact Hartree–Fock^22−24^ (HF) exchange, the localized orbital scaling correction (LOSC),^6^ and range-separation methods.^25^ Moreover, in cases where standard density functionals fail, using the HF density instead of the self-consistent density, known as HF-DFT, has been shown to improve results significantly.^26−33^ For a comprehensive benchmark of HF-DFT, the interested reader is referred to the work of Martin and co-workers.^34^

The good performance of HF-DFT and its appealing theoretical and practical simplicity has led Burke and co-workers to the development of density-corrected HF density functional theory (DC(HF)-DFT).^1,35−44^ Broadly speaking, this method involves two key steps: assessing whether the density generated by the density functional requires correction or replacement and then, *if necessary*, substituting the HF density and evaluating the functional on that density (performing a HF-DFT calculation). This strategy sets DC(HF)-DFT apart from pure HF-DFT, as it ensures—at least in theory—that the HF density is used only when it improves the accuracy of the results. While DC(HF)-DFT has already demonstrated great potential,^35,36,42,43,45−51^ in this work we show that further enhancements are possible.

## Theoretical Considerations

### Why Density-Corrections Might Be Necessary and Useful

The exchange-correlation functional is the only part of (KS-)DFT that is not known exactly and hence needs to be approximated. This approximation is then used twice in common DFT calculations, once when determining the density and again when determining the energy of the system; of course, neither is exact. Despite the name, the accuracy of a certain density functional in terms of energetics does not necessarily guarantee the accuracy of the KS potential or the density itself. In fact, most density functional approximations (DFAs) produce poor KS potentials,^52,53^ which can be seen, e.g., in the poor orbital energies^54^ these functionals yield. Nevertheless, in most cases, the density is still very accurate^40^ because the overall shape of the approximate potential is reasonable, although it is shifted with respect to the exact one, which does not affect the orbitals or the density.^43,44^

However, there are large classes of calculations where the density is poor, leading to significant errors in the calculated energies.^1,36,37,39^ Burke and co-workers developed a framework to distinguish such *density-driven errors* from the errors of the functional itself,^1^ the *functional errors*, by separating the total error according to
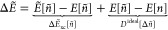
1where exact quantities are denoted without a tilde while approximate quantities are denoted with a tilde symbol; e.g.,  denotes the exact functional evaluated on an approximate density. Since it is impractical to evaluate the exact functional on an approximate density, the following separation was proposed:
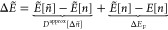
2where the density-driven error (*D*^approx^) is now obtained using an approximate functional *Ẽ*. If the density-driven error exceeds the functional error (Δ*E*_F_), the calculation is considered *abnormal*, which means that the functional itself is (or can be) accurate while the produced density, due to a wrong potential, is poor.^55^ For a more detailed discussion of how this is possible and the underlying theory in general, the reader is referred to ref (1).

Although highly accurate densities can be computed using coupled cluster or configuration interaction approaches, one cannot compute the noninteracting kinetic energies directly from those without performing a KS inversion, which is computationally expensive and numerically challenging.^36,56^ However, it has been shown that for abnormal calculations—contrary to normal calculations, where the functional error dominates—the use of the HF density is, in terms of improving the energetics, often not very different from the use of the exact (or highly accurate) density.^38^ We want to stress that this does not necessarily mean that the HF density is overall better as pointed out by Burke and co-workers several times; it simply means that the density functional evaluated on the HF density shows a smaller density-driven error in these cases.^38^

As previously mentioned, the use of the HF density can be very beneficial; nevertheless, we may not always want to use the HF density. First of all, self-consistency makes the evaluation of properties depending on the derivative of the energy much easier to calculate, since a lot of terms vanish. However, we note that a scheme of calculating gradients for HF-DFT was put forward by Bartlett and co-workers.^32^ Moreover, for normal cases, the self-consistent density usually yields more accurate energetics.^40^ Finally, the HF density should not be employed if it is spin-contaminated since it should no longer be considered more accurate, as pointed out by Burke and co-workers.^57^

### When to Correct the Density

Recently, there has been a vigorous discussion about how to evaluate the accuracy of densities.^37,58−66^ The problem with this is that the density is a function,^37^ meaning that there are infinitely many numbers to compare and hence many ways to do so. Burke and co-workers argued^37^ that the energy is the most meaningful measure since it is the quantity that really matters and it is further able to detect even the tiniest differences in the density *when they matter*, leading to the development of density functional analysis.^38^ The present work deals with more pragmatic but related questions: When is the HF density likely to improve the results obtained with a certain density functional? And how can we do that efficiently?

In order to detect abnormal calculations, Burke and co-workers put forward a simple heuristic called the *density sensitivity* defined as^38^
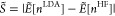
3where *n*^LDA^ and *n*^HF^ denote the LDA and the HF density, respectively. Note that eq 3 represents the density sensitivity of a single calculation, but the density sensitivity is usually evaluated for the whole reaction of interest. If the density sensitivity of this reaction is above a certain threshold (2 kcal/mol is the usual choice^37^) the reaction is considered density sensitive and the HF density is employed instead of the self-consistent density to evaluate the reaction energy.

When comparing eq 3 (the density sensitivity measure) with the ideal density-driven error given in eq 1, it becomes apparent that the former approximates the latter under the following conditions:

- The overall shape of the approximate functional must be accurate. In other words, the energy difference between two points on the approximate energy surface, , should mirror the difference that would be obtained with the exact functional.
- The density obtained from the LDA should be close to the self-consistent density of the approximate functional (denoted by *Ẽ*).
- The HF density should approximate the exact density closely.

These conditions are, of course, rarely met, but this is not very problematic since we are only interested in answering the question of whether the energy calculation is sensitive with respect to the density in use or not. However, there are some weaknesses of the proposed density sensitivity measure, especially in combination with DC(HF)-DFT:

First of all, the density sensitivity is independent of the density generated by the functional being analyzed, although it can differ significantly from the LDA one. Furthermore, when the density sensitivity exceeds a specified threshold, the HF density is *presumed* to be a better choice than the self-consistent density, or even an accurate approximation of the exact density.^42^ This assumption, coupled with the utilization of the LDA density rather than the functional’s self-consistent density, introduces potential difficulties.

Moreover, the density sensitivity is size extensive, which necessitates adjustment of the threshold according to the system size.^35,41^ Additionally, when calculating small energy values such as torsional barrier heights or noncovalent interactions, the threshold must be further adapted,^35,57^ which can introduce an element of arbitrariness.

As mentioned above, the density sensitivity could, in principle, be applied to single calculations, but it is typically used for reaction energies. While it is, of course, true that key chemical concepts are determined by energy differences, this introduces a source of error cancellation.^1^ There is a further source of error cancellation in the density sensitivity measure: since the density sensitivity is measured using an approximate exchange-correlation functional, errors in that functional can cancel the ones in the density as functional errors and density-driven errors have opposite signs.^43^ That such an error cancellation can occur is well-known.^29,56,67^

In that context, we also mention the work of Kepp, who proposed a recipe to assess the degree of normality that evaluates four distinct functionals on each other’s self-consistent densities.^68^ The use of various functionals reduces the probability of error cancellation in measuring the abnormality of the reaction. However, the HF density was not included in this measure, preventing it from detecting a lot of abnormalities. Additionally, for a trial set of *N* functionals, *N*^2^ calculations are necessary for each system, which is computationally demanding.

This leads us to a final issue: the value of DFT lies in its computational efficiency, and this would be significantly reduced if additional HF calculations were to be performed every time. Since the majority of calculations are not density sensitive,^57^ this is a weakness needing to be addressed in order to facilitate more widespread use of DC(HF)-DFT.

In the subsequent discussion, we will try to address the aforementioned weaknesses of the density sensitivity by proposing a novel straightforward and efficient heuristic approach based on the noninteracting kinetic energy for detecting abnormal DFT calculations.

## The Kinetic Energy Indicator

### Theoretical Rationalization

To begin with, let us summarize the key features that an indicator should possess in order to signal the superiority of the HF density for a given DFT calculation, as these characteristics serve as the foundation for our kinetic energy indicator:

First, the indicator should compare, using a specified metric, the *self-consistent density of the specific functional* to the HF density. Second, it should be *size-intensive*; therefore, no adjustment of thresholds should be necessary. Third, it should *avoid error cancellation* as much as possible. Fourth, it should be *efficient*.

Our proposed kinetic energy indicator is very straightforward and requires two calculations: a converged DFT calculation using our preferred density functional and a converged HF calculation on the very same system; it then compares the two (noninteracting) kinetic energies. If the HF kinetic energy is larger than that obtained from the DFT calculation, the HF density is the better choice. But how did we arrive at that conclusion?

We first appeal to the textbook example of a particle confined within a 1D box, where the potential *V*(**r**) is set to zero. In this scenario, the total energy of the particle is solely determined by its kinetic energy. Since the total energy—and hence the kinetic energy—is inverse proportional to *L*^2^, it becomes smaller the larger the box gets.^69^ By extrapolating the insights gained from this simplified example to the problem of delocalization, we can anticipate a similar trend: the kinetic energy of the system will decrease if it undergoes delocalization.

Of course, setting the potential to zero is a crude approximation when it comes to chemical problems. We hence investigated the change in the noninteracting kinetic energy for the slightly more complicated H atom, when we evaluate its electronic structure with different functionals of the form
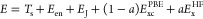
4

In eq 4, *T*_s_ denotes the noninteracting kinetic energy, *E*_en_ denotes the energy stemming from the attraction of the electrons to the nuclei, *E*_J_ denotes the so-called Coulomb energy, *E*_xc_^PBE^ denotes the PBE^70,71^ exchange-correlation (xc) energy, *E*_x_^HF^ denotes the HF exchange energy, and *a* is the mixing parameter, ranging from 0 to 1. Note that we scale the complete PBE xc-energy and so the PBE0^72,73^ functional is not within the set of functionals, but we recover the standard PBE functional for *a* = 0 and the HF functional for *a* = 1.

**Figure 1:**
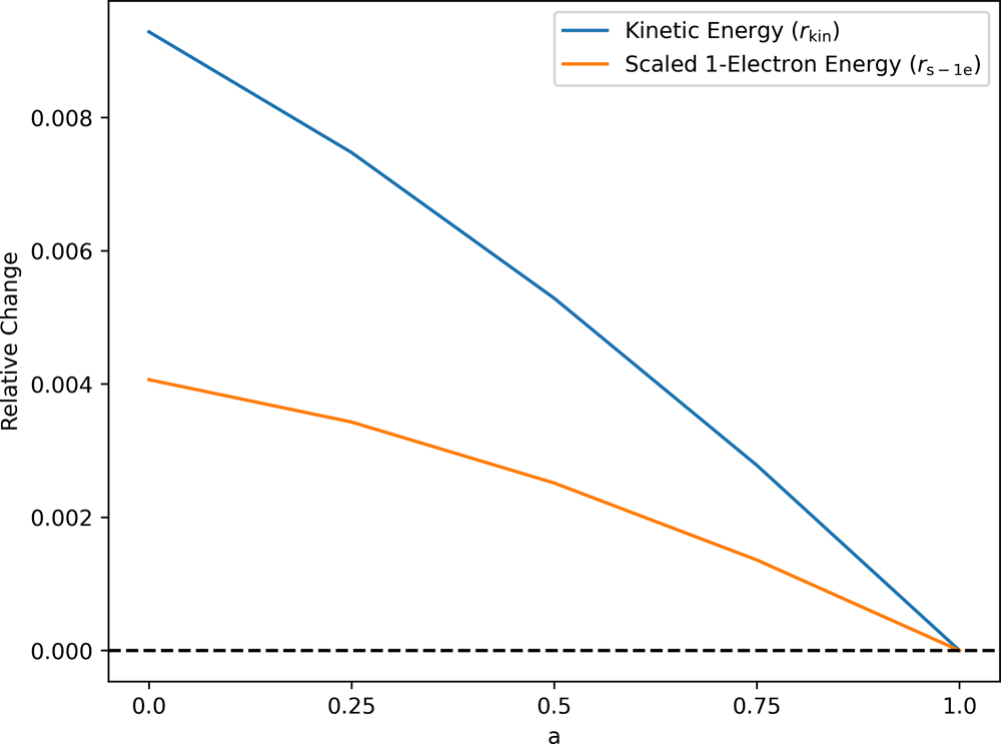
Behavior of the two indicators when interpolating between pure PBE and pure HF (exact) for the H atom. Note that both the exchange and the correlation part of the PBE functional are scaled and hence the functional obtained for mixing factor 0.25 does not correspond to the PBE0 functional.

The relative change of the kinetic energy is given by
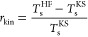
5and is plotted in Figure 1. As can be seen, *r*_kin_ becomes more and more positive as we move from HF (*a* = 1; exact, no delocalization error) to PBE (*a* = 0; delocalization error), meaning that the kinetic energy obtained using the density functional decreases compared to the HF kinetic energy. As reported by Mezei et al., HF can yield quite erroneous densities but which have good gradients and Laplacians of the energy.^58^ We hence consider another indicator, the scaled one-electron energy indicator, to avoid biasing toward derivatives. The scaled one-electron energy indicator is given by
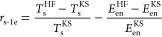
6

The idea behind the scaling is to put equal weight on the density itself and its derivatives. Again, the calculation is considered abnormal if *r*_s-1e_ becomes positive. As can be seen, the scaled one-electron indicator leads to the same conclusions for this simple example.

Thus far, we know that the noninteracting kinetic energy is expected to decrease when evaluated for a delocalized density compared to the exact density. However, in practical scenarios, this knowledge is not particularly useful as the exact noninteracting kinetic energy is, as mentioned above, not easily available. To obtain a more practical measure, we can consider the *virial theorem* in KS-DFT:^74,75^
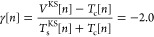
7

For our purpose, the following approximation is reasonable:
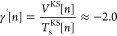
8
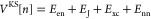
9

Now, since *V*^KS^ incorporates, in contrast to the HF functional, an energy contribution stemming from electron correlation, it seems reasonable to assume that *T*_s_^KS^ should exceed its HF counterpart. We note that this “contraction effect of correlation” was also reported by Baerends and co-workers,^76^ who found that *T*_s_^KS^ > *T*_s_^HF^ holds true for all of their investigated cases.

In this context it should be noted that although the definition in terms of orbitals is identical, the HF and the KS noninteracting kinetic energies are different,^77^ since the HF method minimizes the expectation value of the Hamiltonian over all Slater determinants while the KS Slater determinant can only be constructed from orbitals stemming from a local multiplicative potential yielding the exact density according to
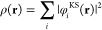
10

However, that difference was shown to be small and this is why it is neglected in DC(HF)-DFT.^36^ It is also true that, contrary to the KS case, no universal proof exists that the HF kinetic energy *needs to be* smaller than (or equal to) the exact (interacting) kinetic energy; or in other words, that *T*_c_ needs to be non-negative. However, a realistic counter example has not been found.^78^

Hence, we reach our final conclusion: The noninteracting kinetic energy obtained from a standard density functional is anticipated to exceed its HF counterpart. If this expectation is not met, it suggests that the underlying self-consistent density is excessively delocalized, and it is recommended to use the HF density instead.

### Sanity Checks on Typical Normal and Abnormal Calculations

To test the kinetic energy indicator, we evaluated it for various DFT calculations on the different systems contained in the S22^79,80^ and B30^81,82^ test sets (a list of all relevant energy contributions for all systems and functionals considered in this work is provided in the Supporting Information), serving as examples for normal and abnormal calculations, respectively.^38^ Both test sets were developed to assess the accuracy of a method in calculating noncovalent interaction energies between molecules and complexes, with high-level coupled-cluster calculations serving as a reference. The well-established S22 test set was designed to represent noncovalent interactions in biological molecules in a balanced way (hydrogen bonds, weak dispersion bonds, and mixed scenarios). On the other hand, the B30 test set contains noncovalent interactions that have been shown to be challenging, particularly for pure density functionals, which produce significant density-driven errors on this test set:^42^ halogen bonds, chalcogen bonds, and pnicogen bonds.^81^ With “systems” we thus mean the various complexes/dimers plus the respective subsystems/monomers.

We used several functionals for our tests: the LDA^83−85^ functional as an example known for large delocalization errors; the PBE^70,71^ and the SCAN^86,87^ functionals since they are probably the most popular nonempirical functionals in use today and SCAN additionally fulfills many exact constraints; and the M06-L^88,89^ functional as an example for a highly empirical functional.

**Figure 2:**
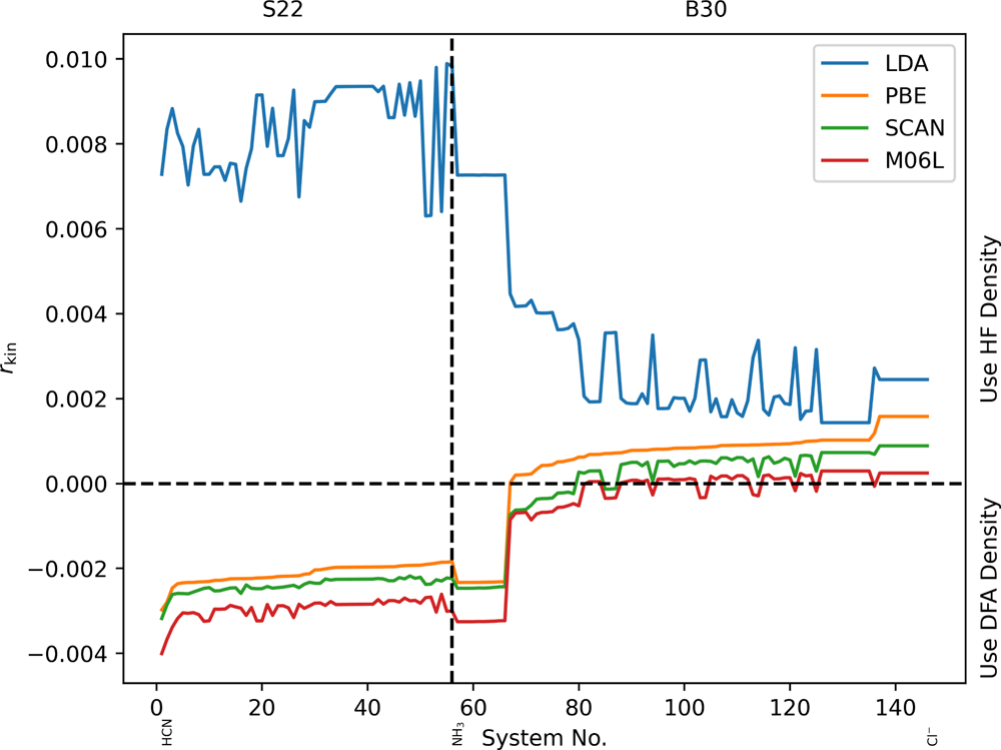
Relative change of the kinetic energy (*r*_kin_) for different DFT calculations on the S22 and B30 test sets.

Figure 2 shows *r*_kin_ as defined above for the different functionals and systems in the S22 and B30 test sets. Note that all systems of a specific test set in this section were ordered by increasing value of *r*_kin_ obtained with the PBE functional; the complete ordered lists can be found in the Supporting Information. As can be seen, for LDA the indicator is always larger than 0 and hence *always* suggests the use of the HF density. For the other three functionals (PBE, SCAN, and M06-L) all calculations in the S22 test set are predicted to be normal, whereas most of the calculations contained in the B30 test set are predicted to be abnormal. Since we chose the B30 test set to represent abnormal DFT calculations, these observations coincide exactly with our expectations. Also note how the indicator changes for different functionals: based on this indicator, the LDA density performs worse, followed by PBE, SCAN, and finally M06-L. Furthermore, the three (m)GGAs seem to produce quite similar densities according to our indicator. While this is interesting to observe, we stress that our indicator is not intended to assess the quality of the different densities but to predict only if the HF density is a better choice for a specific calculation.

**Figure 3:**
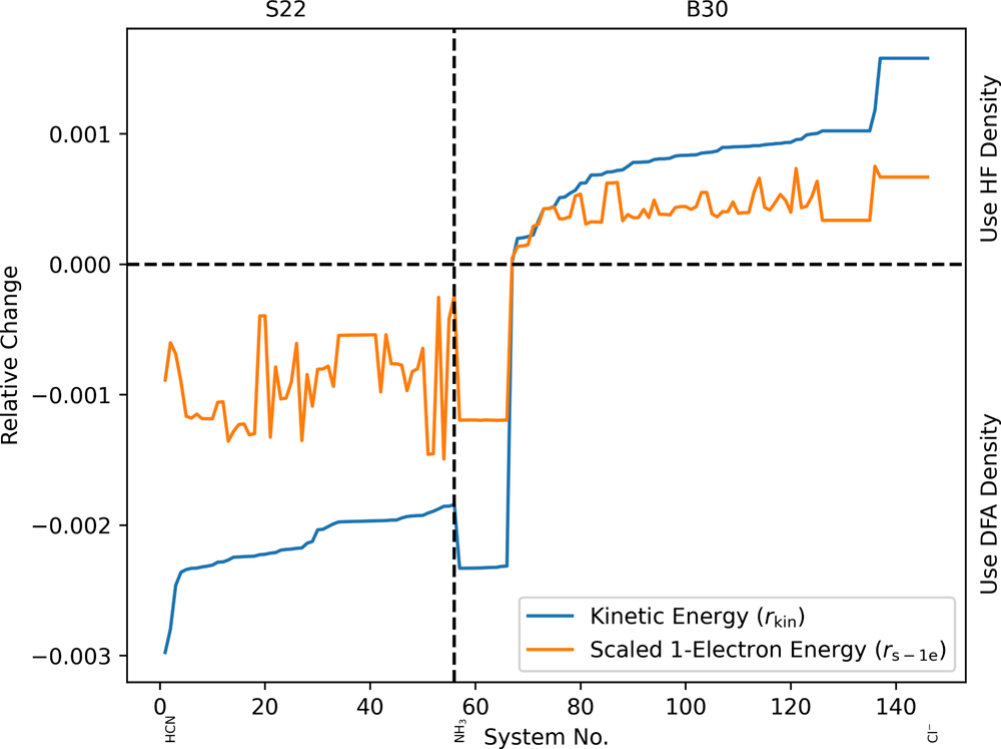
Relative change of the kinetic energy (*r*_kin_) and the scaled one-electron energy (*r*_s-1e_) for the PBE functional on the S22 and B30 test sets.

In Figure 3 we additionally show *r*_kin_ together with *r*_s-1e_ for the PBE functional. As can be seen, as for the H atom, the kinetic energy indicator and the scaled one-electron indicator lead to the same conclusions and hence, we will only use the kinetic energy indicator in the following discussions.

**Figure 4:**
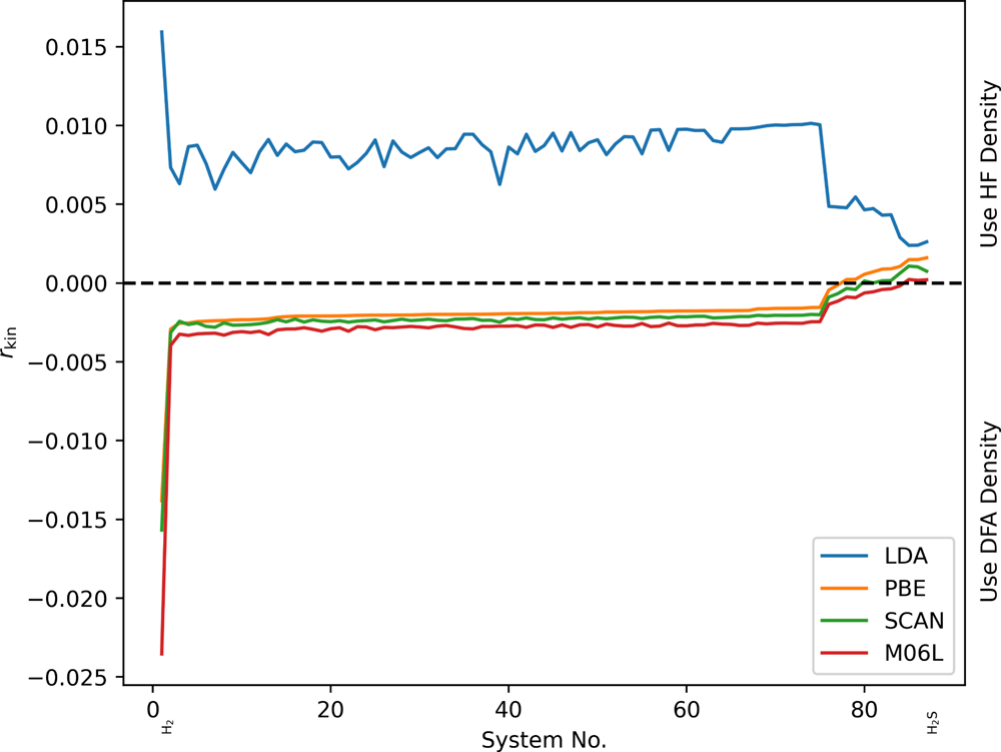
Relative change of the kinetic energy (*r*_kin_) for different DFT calculations on the FH51 test set. Due to convergence problems for some systems, the cc-pVQZ^90−93^ basis set was used for the SCAN functional.

We performed further sanity checks on a test set we would expect to include mostly normal calculations: the FH51 test set^94^ consisting of reaction energies in small inorganic (e.g., NH_3_ or H_2_S; so, no challenging metallic systems or the like) and organic systems. The results are shown in Figure 4.

As can be seen, the results are in line with our expectations: in the case of LDA the HF density should always be the better choice, while the densities produced by the other three functionals should be perfectly normal in the vast majority of cases.

We previously asserted that a key attribute of an indicator, in our view, should be the minimization of error cancellation as far as possible. The two main sources of error cancellation, as identified earlier, include the utilization of an approximate exchange-correlation functional to determine if the HF density should be employed and the consideration of whole reactions rather than single calculations.

Despite the virial theorem that links *V*^KS^ and *T*_s_, we believe that employing the (noninteracting) kinetic energy functional, which is known exactly in terms of orbitals, is a step toward circumventing the first source of error cancellation.

Addressing the second source of error cancellation is straightforward: we deem a reaction abnormal—and hence conduct *all* necessary calculations using the HF density—if *any single* calculation within it is abnormal. Hence, whenever one wishes to evaluate relative energies (energy differences) and one of the calculations exhibits abnormal behavior (*r*_kin_ > 0), all calculations are performed employing the HF density. Note that this is important to ensure consistency and smooth potential energy surfaces.

In the following, we will assess how well this procedure works by benchmarking the accuracies of the resulting DC(HF)-DFT methods for different test sets taken from the GMTKN55 database.^95^ Moreover, we present DC(HF)-DFT results obtained by using Burke’s density sensitivity measure (threshold of 2 kcal/mol) to determine whether to use the self-consistent or the HF density. To distinguish it from the scheme outlined in this work, we refer to this method as DC(HF; *S̃*)-DFT.

### Performance

Let us start with the performance for the noncovalent interaction energies contained in the S22 and the B30 test sets (geometries and reference values for all test sets considered in this work are provided in the Supporting Information). In the last section, it was shown that the kinetic energy indicator always suggests the use of the HF density for LDA. As can be seen in Table 1, this leads to a significant decrease in the mean absolute error (MAE) for both test sets. That the HF density performs better than the LDA density is in line with observations presented in related works.^58,61^ For the other three functionals the conclusion is the same: the kinetic energy indicator suggests the “more accurate” density in both cases; the self-consistent one for the S22 and the HF one for the B30 test set.

**Table 1:** Mean Absolute Errors in kcal/mol of Different Functionals for Different Test Sets

	S22	B30	FH51^b^	G21EA	DARC
LDA	2.18	8.26	6.69	7.83	11.83
LDA@HF	1.35	5.02	5.44	6.92	8.86
DC(HF)-LDA	1.35	5.02	5.44	6.73	8.86
DC(HF; *S̃*)-LDA	1.63	5.33	5.60	6.71	8.86
					
PBE	0.44^a^	2.46	3.44	3.69	6.63
PBE@HF	0.61^a^	1.00	3.44	2.93	7.64
DC(HF)-PBE	0.44^a^	1.00	3.55	3.04	6.63
DC(HF; *S̃*)-PBE	0.44^a^	0.73	3.20	2.69	6.63
					
SCAN	1.16	2.48	2.99	3.37	2.89
SCAN@HF	1.54	0.77	2.56	4.20	3.41
DC(HF)-SCAN	1.16	0.78	2.96	3.29	2.89
DC(HF; *S̃*)-SCAN	1.15	0.96	2.61	3.43	2.89
					
M06-L	0.36^a^	1.34	2.84	3.46	8.15
M06-L@HF	0.61^a^	0.77	2.07	4.13	5.39
DC(HF)-M06-L	0.36^a^	0.86	2.81	3.48	8.15
DC(HF; *S̃*)-M06-L	0.36^a^	0.92	2.12	3.53	5.39

a Corrected using Grimme’s D3 dispersion correction.^99−101^

b Due to convergence problems for some systems, the cc-pVQZ^90−93^ basis set was used for the SCAN functional.

We further tested our kinetic energy indicator for the chemical problems included in the FH51, the G21EA^95−97^ (adiabatic electron affinities), and the DARC^95,97,98^ (Diels–Alder reactions) test sets. Overall, the kinetic energy indicator behaves as desired and leads to significant improvements when the DFT densities are erroneous. However, questions about the reliability of our indicator arise when evaluating the DARC test set using the M06-L functional. In this case, the kinetic energy indicator clearly favors the “less accurate” density. We conducted further examination to understand this behavior better.

**Figure 5:**
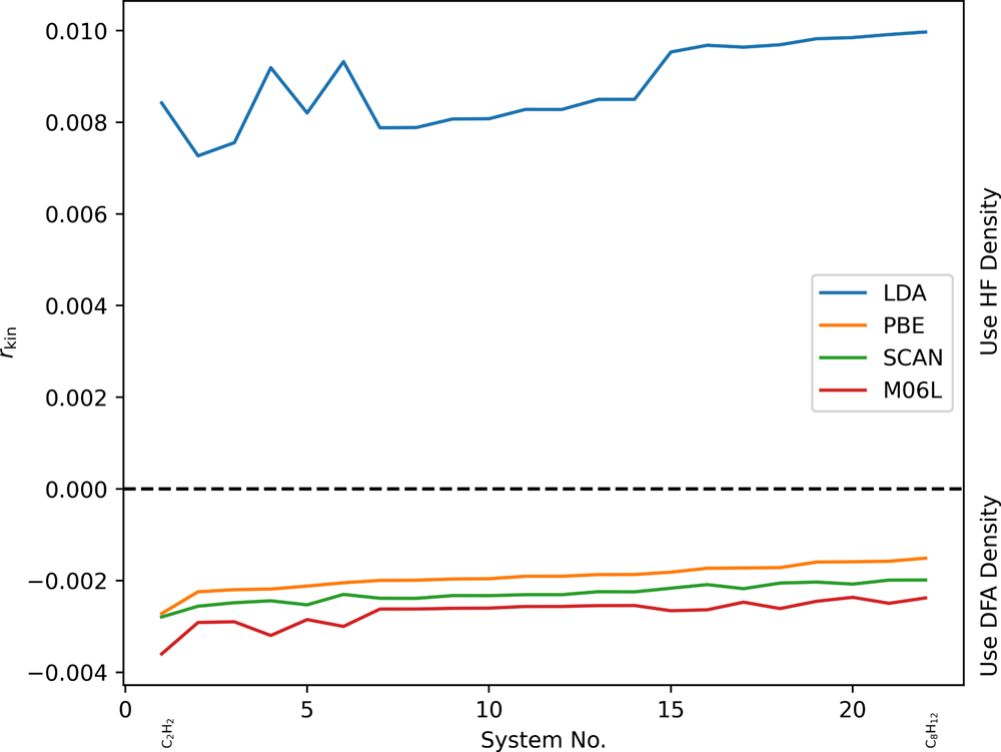
Relative change in the kinetic energy (*r*_kin_) for different DFT calculations on the DARC test set.

**Figure 6:**
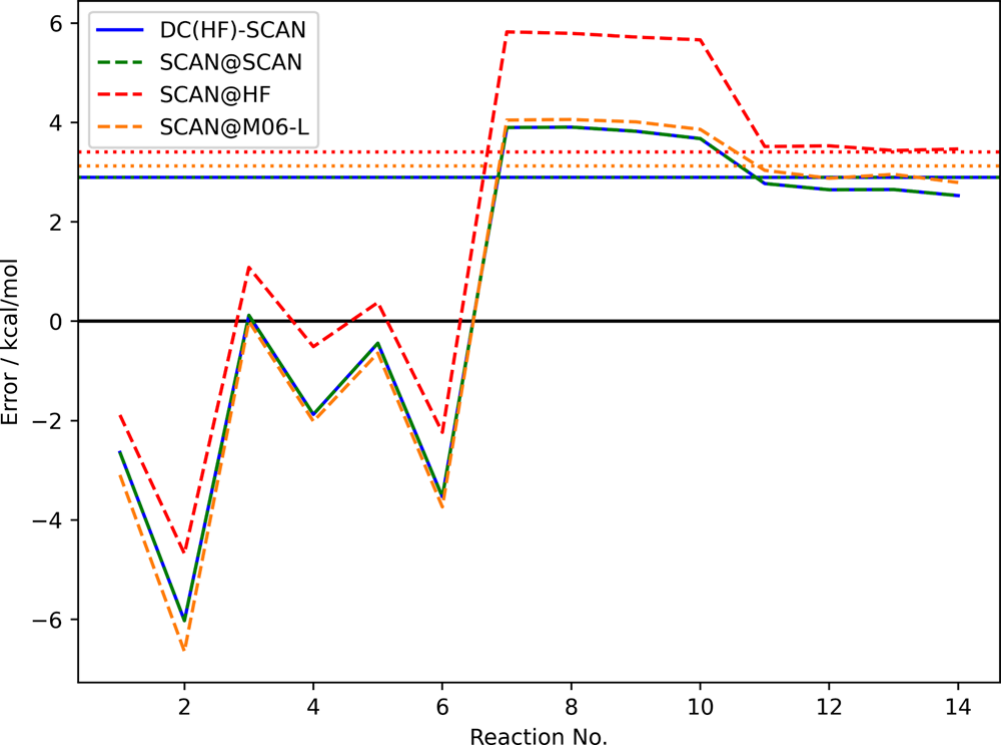
Errors in kcal/mol for the different reactions contained in the DARC test set using the SCAN functional on different densities. The colored horizontal lines show the respective mean absolute errors.

Figure 5 shows *r*_kin_ values for the calculations in the DARC test set performed with the LDA, PBE, SCAN, and M06-L functionals. As can be seen, only for the LDA functional the kinetic energy indicator suggests the use of the HF density. Furthermore, as for the examples presented in the last section, the densities produced by the different (m)GGAs seem to be quite similar (at least according to our kinetic energy indicator). Also note that the kinetic energy indicator performs very well for all functionals except M06-L. Therefore, we tested how the SCAN functional—performing best on the DARC test set—performs when evaluated on the M06-L density. The results are shown in Figure 6.

As can be seen, the errors of the SCAN functional evaluated on its self-consistent density and on the M06-L density are indeed very similar, and hence the kinetic energy indicator correctly predicts the M06-L density to be normal—if the SCAN density is normal then the M06-L density should be normal as well. Therefore, the better performance of M06-L@HF is probably due to a fortuitous cancellation of the functional error and errors in the HF density. Although this behavior of our kinetic energy indicator leads to worse results in this case, it is still encouraging that it is able to make this distinction. We assume similar reasons for the slight worsening of the PBE results for the FH51 test set.

When the DC(HF)-DFT scheme proposed in this work is compared with the one suggested by Burke and colleagues, it is evident that the two schemes exhibit comparable performance overall. The most noticeable difference is seen in the results obtained for the DARC test set using the M06-L functional. In this instance, DC(HF; *S̃*)-DFT mirrors M06-L@HF. As we have previously noted, we believe that the good performance of M06-L@HF is primarily attributed to a fortunate cancellation between the functional error and the errors in the HF density. Although this results in more accurate outcomes in this particular case, these findings highlight the aforementioned potential error cancellations inherent within the density sensitivity measure, an issue that our proposed kinetic energy indicator aims to address.

**Figure 7:**
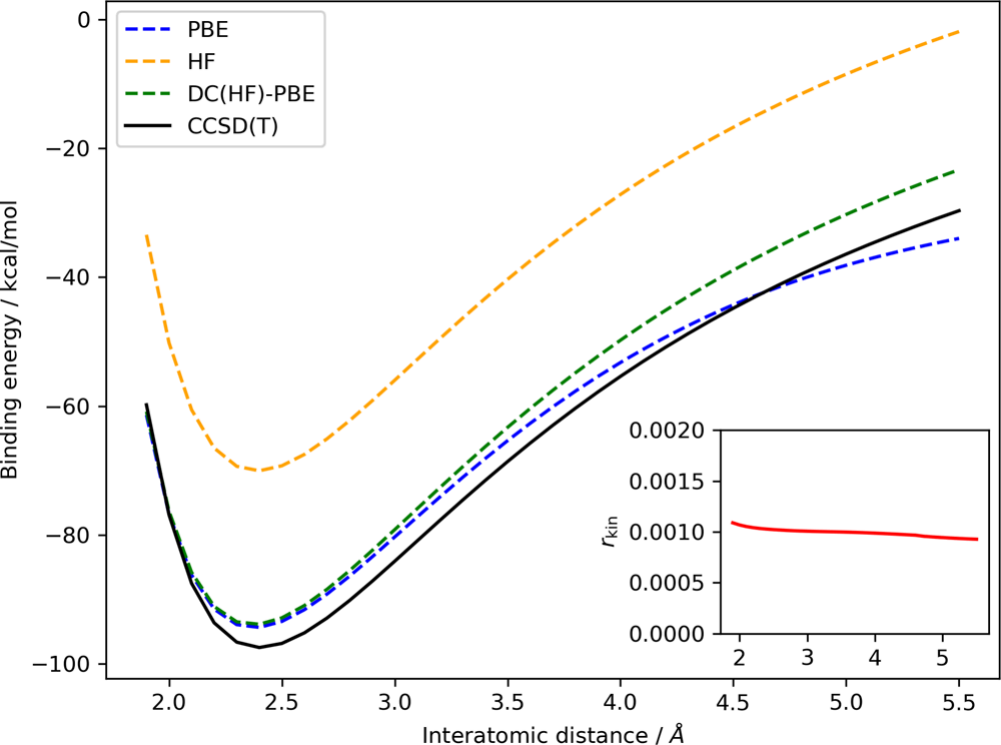
Gas-phase dissociation curve of NaCl, evaluated using PBE, HF, DC(HF)-PBE, and CCSD(T), the latter serving as the reference. The curves are offset by the sum of the respective atomic energies. The inset illustrates the value of *r*_kin_ as a function of the interatomic distance. The *r*_kin_ values for the individual sodium and chlorine atoms are 0.0029 and 0.0004, respectively. Because of convergence issues encountered with the PBE functional for interatomic distances exceeding 5.5 Å, we have restricted our presentation of results to distances up to 5.5 Å.

As a concluding test, we computed the gas-phase dissociation curve of NaCl using several methods: the PBE functional as a representative for a standard density functional, its density-corrected variant, the HF method, and the CCSD(T) method, which acts as a benchmark. Importantly, in the gas phase, NaCl dissociates into the two neutral atoms (homolytic dissociation) rather than the Na^+^ and Cl^–^ ions (heterolytic dissociation) as seen in the aqueous phase. Standard density functionals are known to struggle with this homolytic dissociation, suffering from an incorrect charge transfer, a special type of delocalization error.^19,36,38,46,57^ The dissociation curves, offset by the aggregate energies of the two atoms, are presented in Figure 7.

As illustrated, the PBE functional performs well around the equilibrium distance. However, as the interatomic distance increases (starting at around 4 Å), the PBE functional begins to artificially lower the energy of the dissociating system compared to the accurate dissociation products—the individual atoms. This leads to an incorrect dissociation limit of charged fragments, which is a result of the previously mentioned erroneous electron transfer.

On the other hand, the DC(HF)-PBE curve (shown in green) demonstrates that the density-correction framework, in conjunction with our kinetic energy indicator (depicted in the inset as a function of interatomic distance), successfully rectifies the PBE functional’s incorrect behavior. This results in a dissociation curve nearly shifted by a constant relative to the CCSD(T) reference curve. Also noteworthy is the fact that our kinetic energy indicator suggests the use of the HF density for all interatomic distances. This is in perfect alignment with the findings of Burke and colleagues, who observed that the density-driven error of HF is almost zero for any geometry along the NaCl dissociation curve.^36^

### Efficiency

**Figure 8:**
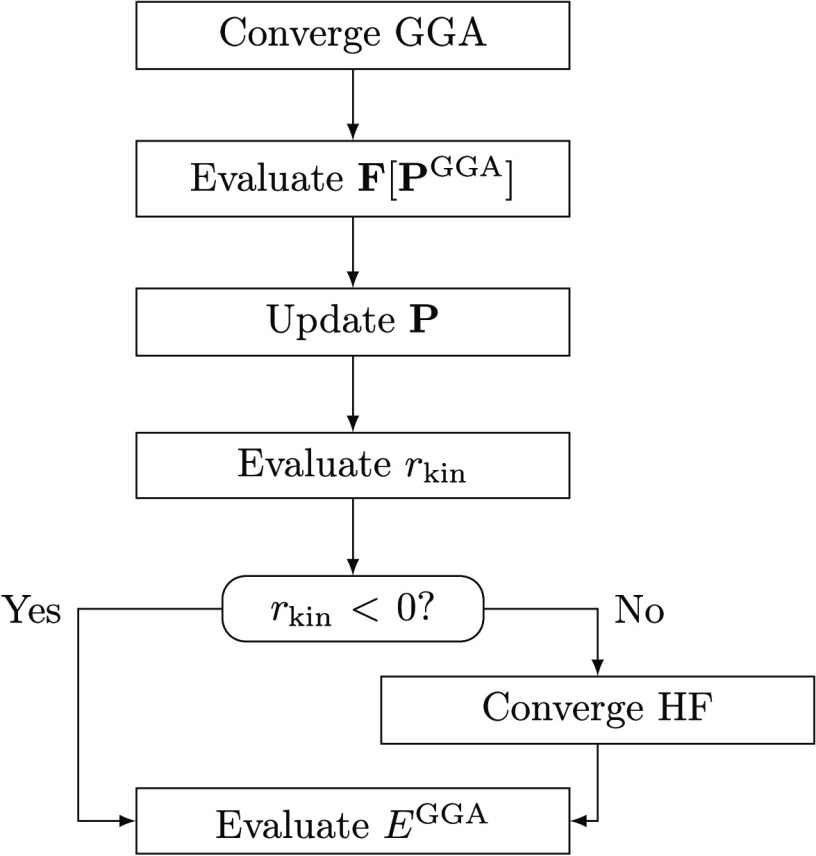
Schematic representation of the efficient DC(HF)-DFT approach.

As mentioned before, another key feature that a good indicator should possess is efficiency. So far, our kinetic energy indicator does not seem to improve much upon the density sensitivity put forward by Burke and co-workers in this respect. In order to address this, we propose the following procedure:

First, converge the DFT calculation. Second, use the converged DFT one-particle density matrix to evaluate one Fock matrix. Third, update the orbitals and density. Fourth, evaluate the kinetic energy using the updated orbitals and compare it with the converged DFT kinetic energy; only converge the HF calculation if *T*_s_^HF,1-iter^ > *T*_s_^KS^. A schematic representation of the approach is depicted in Figure 8.

**Figure 9:**
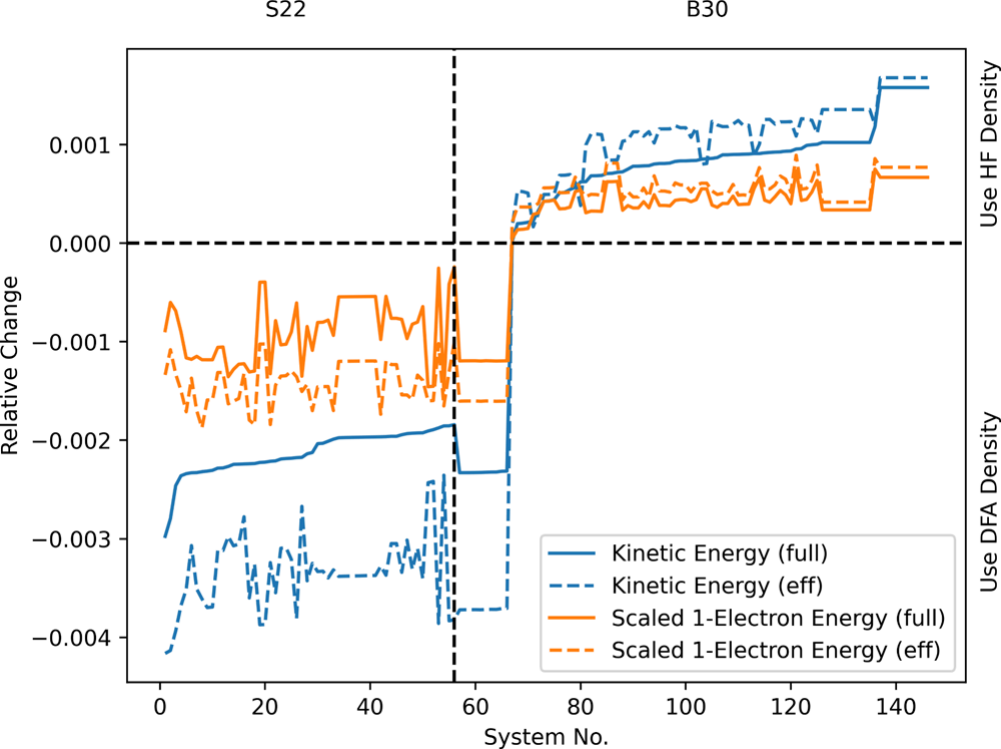
Comparison of the two indicators evaluated with unconverged HF orbitals (only one HF iteration; eff) with their converged counterparts (full).

We investigated that scheme for the S22 and B30 test sets using the PBE functional. The indicators *r*_kin_ and *r*_s-1e_ after only one HF iteration (denoted with “eff”) and the converged counterparts (denoted with “full”) are shown in Figure 9. As can be seen, the indicators after only one HF iteration lead to the same results. Moreover, the unconverged indicators tend to be larger in magnitude, which is ideal since it ensures correct predictions.

**Figure 10:**
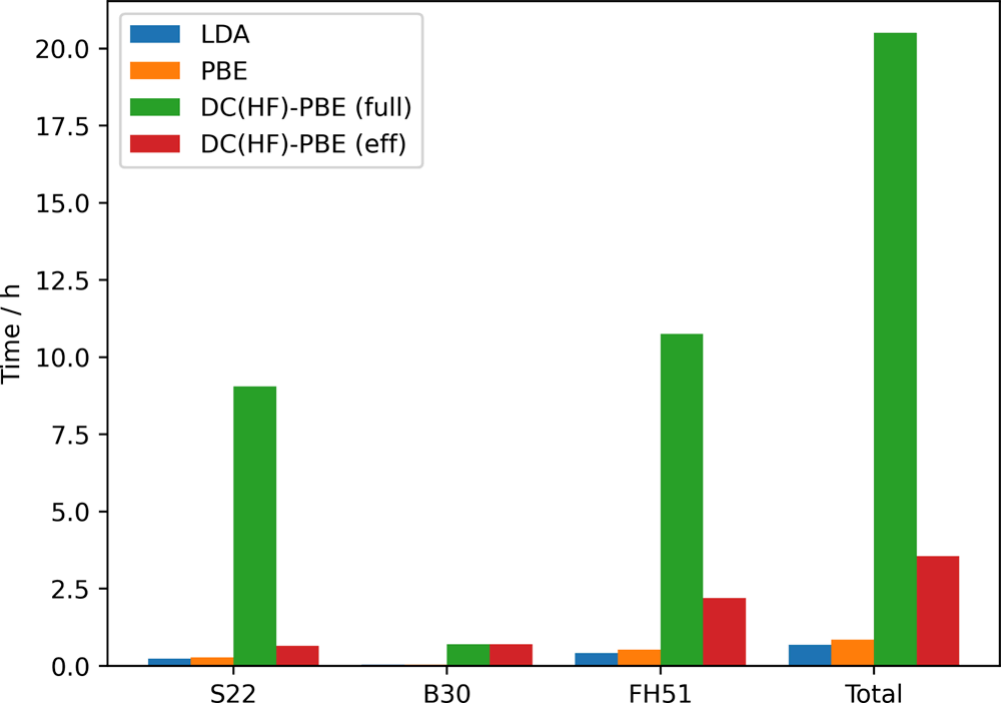
Cumulative timings for the S22, B30, and FH51 test sets.

Figure 10 shows cumulative timings of pure LDA and PBE, as well as full DC(HF)-PBE (converging the HF calculation to assess whether the HF or the self-consistent density should be used) and efficient DC(HF)-PBE (only one iteration of HF for the assessment) for the S22, B30, and FH51 test sets; additionally, the time needed for all test sets together is shown. The reason we also show LDA timings is the fact that the LDA and the HF density are needed to evaluate the density sensitivity according to eq 3.

To start, we note that the HF calculations are significantly more expensive than both the LDA and the PBE calculations; in fact, the difference between the LDA and PBE is negligible. Second, the savings in terms of computational cost are enormous when our efficient DC(HF)-PBE method is employed and, of course, get even larger when more normal calculations are included. We want to stress again that the vast majority of DFT calculations is normal, and hence our proposed procedure is an important step to make the use of DC(HF)-DFT more routine.

Finally, although it was not necessary in the cases investigated here, it should be noted that if the efficient indicator suggests the use of the HF density, it is, of course, possible and probably also advisable to check the indicator again after the HF calculation is fully converged; there is no disadvantage in doing that. Additionally, the density sensitivity could be evaluated with a small extra cost to introduce a further control mechanism. In that way, the density sensitivity and the kinetic energy indicator can be considered complementary.

### Beyond Density Corrections

**Figure 11:**
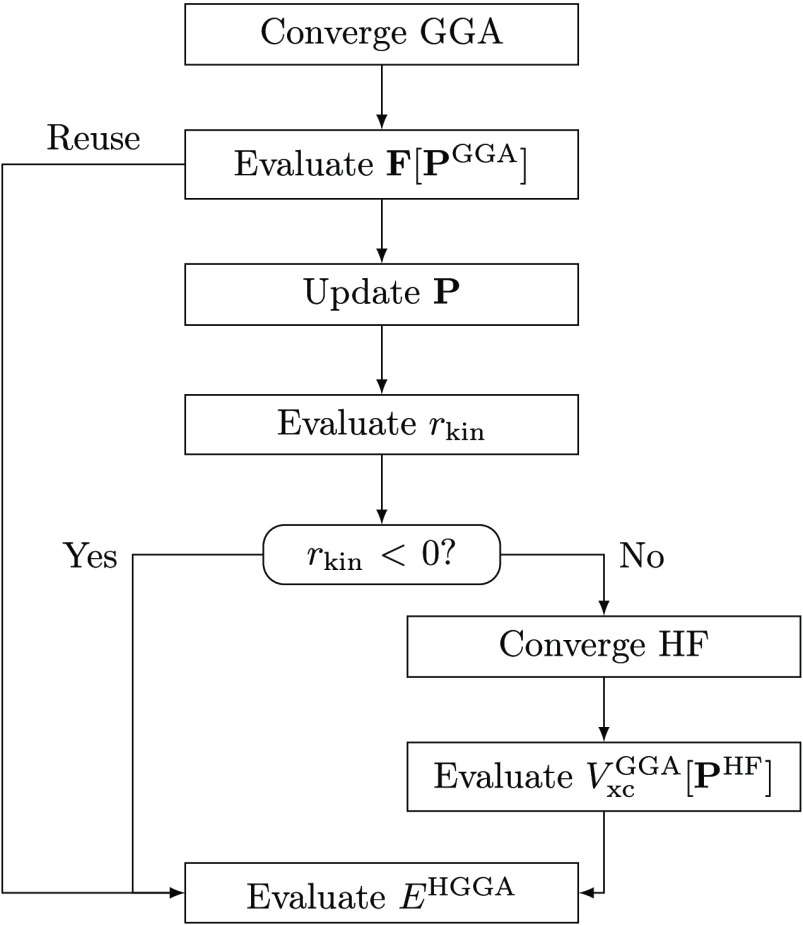
Schematic representation of the C(HF)-DFT approach.

In the previous section, we proposed a scheme that significantly improves the efficiency of our kinetic energy indicator. In this section, we want to go one step further: since our indicator necessitates one iteration of HF in any case, it naturally lends itself to including exact (HF) exchange in the final energy and, in that way, additionally “correcting” the *functional*. Consider the PBE functional as an example:

First, we converge a PBE calculation. After that, we use the PBE one-particle density matrix to evaluate the Fock matrix, update the orbitals, and compare the updated kinetic energy with that obtained using the PBE functional. If the PBE kinetic energy is larger, then we already have everything we need to evaluate the PBE0 functional on the PBE density. If the updated kinetic energy is larger, we converge the HF calculation and the only thing we need to do now is to evaluate the PBE exchange-correlation potential using the HF density on top of that, which is, as can be seen in Figure 10, almost negligible in terms of computational cost. We note that the choice of hybrid to evaluate in this step is completely flexible. Instead of DC(HF)-DFT we call this procedure C(HF)-DFT (“corrected” instead of “density corrected”), or for the specific case of PBE, C(HF)-PBE. A schematic representation of the approach is shown in Figure 11.

We tested the proposed method on the test sets already used above. The results are shown in Table 2. As can be seen, C(HF)-PBE significantly improves upon pure PBE and performs similarly to full (pure) PBE0. It is also worth noting the improvement of C(HF)-PBE compared with PBE0 for the B30 test set, which is due to the use of the HF density in that case.

**Table 2:** Mean Absolute Errors in kcal/mol of Different Functionals for Different Test Sets

	S22	B30	FH51	G21EA	DARC
PBE	0.44^a^	2.46	3.44	3.69	6.63
DC(HF)-PBE	0.44^a^	1.00	3.55	3.04	6.63
C(HF)-PBE	0.45^a^	0.90	2.62	2.59	3.08
PBE0	0.46^a^	1.62	2.63	2.53	3.05

a Corrected using Grimme’s D3 dispersion correction.^99−101^

## Computational Details

All calculations were carried out using a development version of the FermiONs++ program package developed in the Ochsenfeld group.^102−104^ The binary has been compiled with the GNU Compiler Collection (GCC) version 12.1. The calculations were executed on a compute node containing 2 Intel Xeon E5-2630 v4 CPUs (20 cores/40 threads; 2.20 GHz). All runtimes given are wall times, not CPU times.

The evaluations of the exchange-correlation terms were performed using the multigrids defined in ref (105) (smaller grid within the SCF optimization and larger grid for the final energy evaluation), generated with the modified Becke weighting scheme.^105^ The SCF convergence threshold was set to 1 × 10^–6^ for the norm of the difference density matrix ∥Δ**P**∥.

We employ the integral-direct resolution-of-the-identity Coulomb (RI-J) method of Kussmann et al.^106^ for the evaluation of the Coulomb matrices and the linear-scaling seminumerical exact exchange (sn-LinK) method of Laqua et al.^107^ for the evaluation of the exact exchange matrices.

For computations involving the H atom, we utilized the def2-QZVPPD^108−110^ basis set, coupled with the def2-universal-JFIT^111^ auxiliary basis set for RI-J. For the NaCl dissociation, we used the def2-QZVPPD basis set; no RI-J was employed. The reference CCSD(T) computations were carried out employing the Q-Chem^112^ software package and an identical basis set. Unless otherwise stated, all calculations included in the S22, B30, FH51, G21EA, and DARC test sets were conducted using the aug-cc-pVQZ^90−93,113^ basis set in conjunction with the cc-pVTZ-JKFIT^114^ auxiliary basis set for RI-J.

## Conclusion

In conclusion, we presented a straightforward, yet efficient, procedure to perform DC(HF)-DFT calculations. In this procedure, the crucial step of deciding whether the self-consistent or the HF density should be used to evaluate the density functional is conducted employing a straightforward heuristic based on the difference between the noninteracting kinetic energies obtained from the analyzed functional and the HF method, called the kinetic energy indicator. Our kinetic energy indicator offers several key characteristics that make it stand out from other methods: First, it directly compares the self-consistent density of the analyzed functional with the HF density. Second, it is size-intensive, meaning that it is suitable for use in both large and small systems. Third, it reduces the probability of error cancellation, making it more reliable. Finally, it is highly efficient. We further note that our kinetic energy indicator is extremely straightforward to apply in a retrospective analysis of DFT calculations, assuming that the noninteracting kinetic energies of the analyzed DFT calculations are known. All that is necessary is to converge a HF calculation and compare the two noninteracting kinetic energies.

It was shown that the kinetic energy indicator reliably detects calculations where the use of the HF density leads to improved results. Furthermore, the high efficiency of our indicator was demonstrated on three different test sets contained in the GMTKN55 database.

In addition, we have introduced a new procedure, called C(HF)-DFT, which not only corrects the density if necessary but also “corrects” the functional by evaluating a related hybrid at almost no extra computational cost. We have demonstrated its effectiveness using the PBE functional, showing a significant improvement in accuracy that is comparable to that of its parent hybrid, PBE0. Additionally, if the parent hybrid suffers from a density-driven error, then C(HF)-DFT can achieve even higher accuracy. Extending this procedure to double hybrids is work in progress.

Overall, our presented methods provide simple and effective solutions for improving density functional evaluations. As Burke and co-workers have noted,^67^ even small improvements in our current density functional approximations can have a significant impact on applications in science and technology. Therefore, we hope that our contributions will lead to more widespread application of DC(HF)-DFT and C(HF)-DFT, and in that way have a positive impact on quantum chemical applications of all kinds.

## Supporting Information Available

Excel file containing all relevant energy contributions for all systems and functionals considered in this work (XLSX)
Text file containing the ordered lists of systems for the S22, B30, FH51, and DARC test sets (TXT)
Geometries and reference values for the S22, B30, FH51, G21EA, and DARC test sets (ZIP)
